# Histamine and Delirium: Current Opinion

**DOI:** 10.3389/fphar.2019.00299

**Published:** 2019-04-09

**Authors:** Paul L. Chazot, Laura Johnston, Edel Mcauley, Stephen Bonner

**Affiliations:** ^1^Department of Biosciences, Durham University, Durham, United Kingdom; ^2^Intensive Care, South Tees Hospitals NHS Foundation Trust, The James Cook University Hospital, Middlesbrough, United Kingdom

**Keywords:** histamine, H3 receptor, delirium, CNS, autoreceptor, heteroreceptor

## Abstract

Delirium is a very common, but refractory clinical state, notably present in intensive care and in the growing aging community. It is characterized by fluctuating disturbances in a number of key behavioral features, namely cognition, mood, attention, arousal, and self-awareness. Histamine is arguably the most pleotropic neurotransmitter in the human brain, and this review provides a rationale, and proposes that this neuroactive amine plays a role in modulating the characteristic features of delirium. While centrally permeable H_1_ and H_2_ histamine receptor antagonists have pro-delirium potential, we propose that centrally permeable H_3_ histamine receptor antagonists may provide an exciting new strategy to combat delirium. The Histamine H_4_ receptor may also have an indirect inflammatory neuroglial role which requires further exploration.

## Introduction

### What Is Delirium?

Delirium is a very common, but refractory clinical state, notably commonly present in intensive care and in the growing aging community, with occurrence rates ranging from 14 to 56%, and hospital mortality rates ranging from 25 to 33% (Leslie and Inouye, 2011). It is characterized by fluctuating disturbances in arousal, attention, cognition, mood, and self-awareness, which can arise acutely, either in the absence of prior intellectual impairment or superimposed on chronic intellectual impairment in the growing aging population. The rise and development of delirium has been associated with increased morbidity, persistent functional decline, increased nursing time, higher hospital costs, increased length of hospital stay, and higher rates of nursing car home placement. Worryingly, delirium is a common, serious, and refractory source of mortality in intensive and community care across the age range, but is only recently being addressed in the United Kingdom, Europe and worldwide (Leslie and Inouye, 2011). Delirium in older hospitalized patients is of particular concern because patients aged 65 years and over currently account for more than half of all days spent in hospital care.

Delirium is a neurobehavioral syndrome caused by dysregulation of neuronal activity often secondary to serious systemic disturbances. Over time, a number of theories have been proposed in an attempt to explain the processes leading to the development of delirium (Maldonado, 2015; Herling et al., 2018). Each proposed complementary theory has focused on combinations of specific mechanisms or pathological processes (e.g., dopamine excess or acetylcholine deficiency, inflammatory responses), observational qualitative evidence (e.g., sleep deprivation, aging), or empirical scientific data (e.g., specific pharmacological agents such as opioids) or intraoperative hypoxia state association with postoperative delirium) (Maldonado, 2013; Egberts et al., 2018). The literature suggests that many factors or mechanisms included in these theories lead to a final common outcome associated with an alteration in neurotransmitter synthesis, function, and/or availability that triggers the complex behavioral and cognitive changes reported in delirium. In general, the most commonly described neurochemical changes associated with delirium include deficiencies in acetylcholine and/or melatonin, together with excess in glutamate and monoamines dopamine and noradrenalin, and bi-directional activity alterations (e.g., decreased or increased activity, depending on delirium presentation and trigger) in serotonin, γ-aminobutyric acid (GABA) and/or, importantly, histamine (Maldonado, 2013). The unknown nature of etiology for most types of delirium and the complete lack of placebo-controlled Randomized Controlled drug Trials, the lack of any FDA-approved drug treatment for delirium and the wide ranging nature of drugs with multiple chemical neurotransmitter pathways affected (variable across NHS Trust hospitals) used to treat it is clearly a major problem. Furthermore the lack of effective non-pharmacological approaches is also problematical (Wade et al., 2015, 2019; Richards-Belle et al., 2018) Without understanding more about the underlying nature of the pathways involved how can we hope to effectively and rationally treat it?

In this short commentary, we offer a rationale for a new pharmacological strategy to combat delirium. We propose that central histamine is a significant player in all of the clinical features of delirium; while H_1_ and H_2_ histamine-targeted antihistamines should be treated with care, a centrally acting histamine H_3_ receptor antagonist, with appropriate diurnal pharmacokinetic properties, may provide a novel and effective strategy for preventing or combatting delirium. We discuss the key evidence base and potential mechanisms underpinning these proposals and clinical implications.

## Histamine and Anatomical Framework Relevance to Delirium Cognition, Mood and Wakefullness

Arousal stems from the wakefulness of a person and awareness is the individual’s ability to perceive his/her environment. In both these behavioral states, histamine has a primary role to play. Diminished alertness, delayed reaction times, and somnolence are common manifestations of allergy treatments with use of classic first-generation (CNS-permeant) anti-histamines, thus evidencing that histamine is required for arousal/wakefulness and awareness/attention. The evidence became stronger with the report that histidine decarboxylase (HDC) knockout mice, which lack histamine, display increased paradoxical sleep, sleep-wake cycle modifications, and are unable to remain awake under diurnal high vigilance (narcolepsy) (Parmentier et al., 2002). The mammalian, including human, waking state is maintained by continual activation of neuromodulatory aminergic neurotransmitters [dopamine, noradrenaline (NA), acetylcholine and notably histamine], hypocretin/orexinergic (Oxergic), and selective excitatory glutamatergic and inhibitory GABAergic pathways (Korotkova et al., 2005; Yu et al., 2018). Cortical activation is one of the physiological signs of wakefulness and requires robust cholinergic, noradrenergic, serotonergic and, importantly, histaminergic tones. Histamine controls these features through the extensive influence of ascending branches from the tuberomammillary nucleus (TMN) in the hypothalamus to all parts of the brain, including the prefrontal cerebral cortex, various limbic regions and the basal ganglia (Yu et al., 2018). Monoaminergic neurons comprising noradrenergic (locus coeruleus: LC), serotonergic (raphe nuclei, RN) and histaminergic (TMN) neurons project to the cerebrocortex, thalamus and brainstem are known together as the center of sleep regulation (Oh et al., 2018; Yu et al., 2018). Furthermore, Oxergic cell bodies in the hypothalamus densely project to LC, TM and RN, which suggests a strong link between monoaminergic and Oxergic neurons, again in the control of wakefulness. It is well established that the Oxergic wake-active neurons provide a major excitatory drive onto TMN histamine neurons (Lin et al., 2011), and this could be a key way that orexin promotes arousal, through amplifying its effects via the histaminergic system. During the wake state, TMN histamine/GABA-ergic neurons are co-active in parallel and their ascending histamine/GABA fibers release histamine and GABA into the prefrontal cortex (PFC), neocortex (Ctx) and striatum (Str) (Lin et al., 2011). Glutamatergic pyramidal neurons in the PFC send excitatory projections back to the histamine neurons in the TMN, reinforce wakefulness, attention and consciousness (Lin et al., 2011). The Histamine-GABA-ergic neurons are silenced during Non-REM sleep by preoptic GABAergic neurons. Histamine-only projections from the TMN also excite cholinergic neurons into the basal forebrain, and the axons of these excited cholinergic neurons release acetylcholine throughout the cortex (the fundamental basis for high attention together with productive cognitive function). We suggest that when the brain is exposed to these neuromodulators appropriately, we are wakeful, attentive and conscious, importantly, *without* delirium. This this overall neuronal anatomical framework provides the mechanistic basis for the influence of histamine upon delirium, through auto- and hetero-presynaptic and postsynaptic functions, respectively.

## Histamine Receptor Subtype-Dependent Effects

**FIGURE 1:**
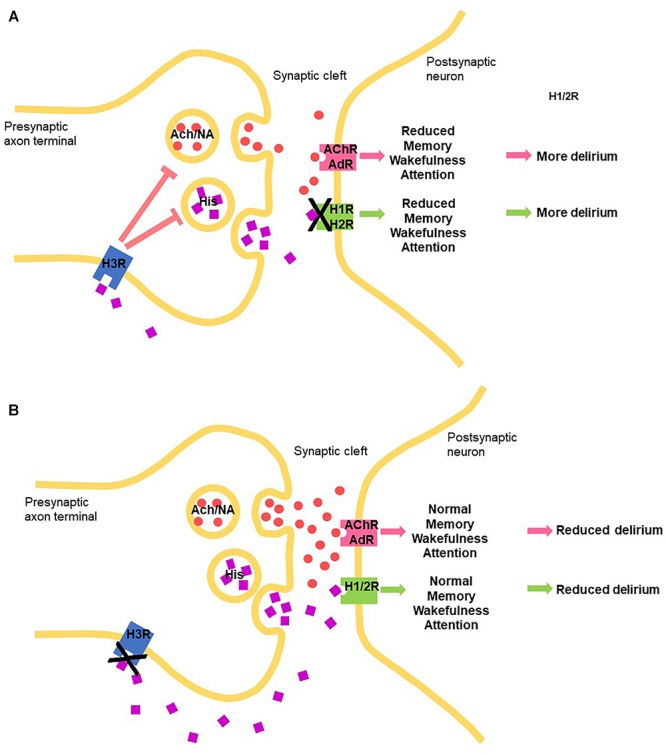
Schematic how manipulating the central histaminergic system by blocking the histamine receptors can potentially modify delirium symptom induction and maintenance through manipulating levels of histamine (HIS) through autoreceptors, acetylcholine (Ach) and noradrenalin (NA), through heteroreceptors binding to histamine receptors, acetylcholine receptor (AChR) and AdR (adrenergic receptors, respectively. **(A)** H_1_ and H_2_ histamine receptor antagonists. **(B)** H_3_ receptor antagonists.

Histamine elicits its physiological action via four G-protein couple receptor (GPCR subtypes, namely H_1_, H_2_, H_3_, and H_4_ receptors, expressed widely and differentially throughout the body, including the CNS (reviewed in Panula et al., 2015). In terms of the brain, H_1_, H_2_, H_3_ receptors play clear roles in neuronal function, post- and pre-synaptically, and, interestingly, the H_4_R appears to influence neuronal function indirectly through modulating activated microglia (Zhou et al., 2018). Histamine driven H_1_ and H_2_ receptor-mediated actions are mostly excitatory, while H_3_ receptors act as inhibitory auto- and heteroreceptors (Panula et al., 2015). Histamine-mediated excitation was blocked by a CNS-permeable H_1_ receptor antagonist, mepyramine, in 78% of cells and by cimetidine, a CNS-permeable H_2_ receptor antagonist, in 42% of cells (Korotkova et al., 2005). Histamine H_3_ heteroreceptor function modulates cholinergic, GABA-ergic, as well as noradrenergic function (Panula et al., 2015) (Figure 1).

## Histamine H_1_ Receptor Cns Physiology

Histamine H_1_ receptors occur throughout the CNS, with particular high densities in regions involved in arousal and waking, notably the thalamus and cortex, and neurochemically the cholinergic, noradrenergic, dopaminergic, and serotonergic nuclei. H_1_ receptor activation causes excitation in many brain regions (brain stem, thalamus, hypothalamus, cortex, amygdala, striatum) through G_q11_ protein and direct block of leak K^+^ conductance or phospholipase C, inositol trisphosphate (IP_3_), and diacylglycerol (DAG) mediation (discussed in Obara et al., 2019). IP_3_ releases Ca^2+^ from internal stores and activates a number of Ca^2+^-dependent processes, including the opening of a cation channel of the transient receptor potential canonical (TRPC) type or stimulation of a Na^+^–Ca^2+^-exchanger. Furthermore, the elevated intracellular Ca^2+^ can stimulate NO synthase and, consequently, guanylate cyclase. On the other hand, Ca^2+^-dependent K^+^ channels can be opened, leading to hyperpolarization and inhibition, for instance, in hippocampal pyramidal neurons.

## H_2_ Histamine Receptor Cns Physiology

Histamine H_2_ receptors are also widely distributed in the mammalian brain (reviewed in Panula et al., 2015). The highest densities of histamine H_2_ receptors are found in the basal ganglia, hippocampus, amygdala and cerebral cortex, with modest expression levels in the cerebellum and hypothalamus (Panula et al., 2015; Monczor and Fernandez, 2016). A similar distribution of the histamine H_2_ receptor occurs in the brain of humans and rodents. H_2_ histamine receptor antagonists decreased significantly the hypothalamic NA content by 21–32%. Activation of the histamine H_2_ receptor in the brain inhibits nerve cells and blocks long-lasting afterhyperpolarization and accommodation of firing in cortical and thalamic neurons (Haas and Reiner, 1988). However, if this afterhyperpolarization block continues for an protracted period, it can lead to potentiation of excitation in rodent and in human brain, resulting in enhanced synaptic plasticity (Brown et al., 1995). Therefore, H_2_ receptor antagonism can suppress plasticity. RT-PCR revealed that while mRNA for the H_1_ receptor was expressed in 77% of isolated LC neurons, mRNA for the H_2_ receptor was in 41% and H_3_ receptors in 29% of LC neurons. These findings underline the coordination between aminergic systems and suggest that the arousal induced by the histamine system could involve excitation of noradrenergic neurons in the LC (Korotkova et al., 2005).

## H_3_ Receptors Cns Physiology

H_3_ receptors, pre-synaptic inhibitory GPCRs, inhibit voltage-activated Ca^2+^ channels, on the terminals of histaminergic axons themselves (as an autoreceptor) and many types of neurons (heteroreceptor), which leads to reduced transmitter release of histamine, and acetylcholine, noradrenalin, serotonin, GABA, glutamate (heteroreceptors), respectively (reviewed in Panula et al., 2015). Despite H_3_ receptors being predominantly presynaptic receptors, regulating the release of neurotransmitters such as acetylcholine and histamine in most areas of the brain, in a particular part of the brain, namely the striatum, the vast majority of these receptors are actually postsynaptic, affecting signaling throughout the basal ganglia. Because the basal ganglia are centrally involved in several major neurological and psychiatric disorders, this aspect requires consideration. Constitutive activity *in vivo* and the possibility of dimerization shown *in vitro* for the H_3_ receptor has been reported (summarized in Panula et al., 2015), but relevance to physiological function of these functional and structural features and, therefore, to modulating delirium, is unclear.

## H_4_ Receptors Cns Physiology

A number of recent experimental studies suggest that systemic inflammation contributes to the pathophysiology of delirium in both elderly and post-trauma delirium. A common raised inflammatory cytokine linked to delirium in these studies is IL-6. Histamine via the histamine H_4_ receptor is known to play a key role in activating systemic inflammation through activation of microglia, mast cells and immune dendritic cells, with consequent production of proinflammatory factors TNF-α and, notably, IL-6 (Desai and Thurmond, 2011; Simon et al., 2011; Vasunilashorn et al., 2015; Ngo et al., 2017; Zhou et al., 2018). A rise in microglia H_4_R has been implicated in Parkinson’s disease, in which delirium has been a recently recognized feature. This offers a possible role for the H_4_R in the neuroinflammatory components of delirium.

## Histamine Receptor Subtype Connections in the Delirium Clinical Setting and Wakefullness

### Histamine H_1_ and H_2_ Receptors

Drug-induced delirium is often seen in clinical practice. Even before it was discovered that histamine was a transmitter in the brain, first generation anti-histamines (i.e., H_1_ receptor antagonists) were noted historically to be sedatives (eg., Monnier et al., 1967). Interestingly, H_1_ receptor antagonists, for example, doxepin (at low concentrations), are making a comeback to treat primary insomnia (Yeung et al., 2015). Histamine and H_1_Rs are involved in maintaining arousal and cognition in humans, and that the severity of clinical symptoms is correlated to the amount of antihistamine that has penetrated into the brain (Tashiro et al., 2002). It was noted, as far back as the 1980s, that delirium was a rare side-effect of both H_1_ and H_2_ antagonists (reviewed in Yanai et al., 2017) (Table 1). First generation H_1_ anti-histamines significantly increased daytime sleepiness and nocturnal sleep quality. Some, including cetirizine and hydroxyzine, seemed to also have negative influences on mood states. Outpatients who received cetirizine and hydroxyzine treatments reported higher scores on the depression, anxiety, and fatigue sub-scales compared to those who received desloratadine, levocetirizine, and rupatadine (Clegg and Young, 2011). The sedating antihistamines are non-specific in their actions and often have marked anticholinergic effects. Features of overdose include tachycardia, blood pressure disturbances, dry mouth, ataxia, psychosis, convulsion and, notably, agitation (Clegg and Young, 2011).

**Table 1:** The implication of histaminergic system and drugs to delirium.

Drug	Selectivity	CNS-permeable?	Effects on delirium?	Reference
Doxepin	H_1_	Yes	unknown	Yeung et al., 2015
Cetirizine	H_1_	Yes	possible	Clegg and Young, 2011
Hydroxyzine	H_1_	Yes	possible	Clegg and Young, 2011
Desloratadine	H_1_	No	No	Clegg and Young, 2011
Levocetirizine	H_1_	No	No	Clegg and Young, 2011
Rupatadine	H_1_	No	No	Clegg and Young, 2011
Betahistine	H_1_	No	Mixed data	Hoenders and Wilterdink, 2004
Cyproheptadine	H_1_	Yes	Decreased incidence but not severity	Page et al., 2009
Cimetidine	H_2_	Yes	Possible	Cantú and Korek, 1991
Cimetidine	H_2_	Yes	Yes	Nowak, 1980
Cimetidine	H_2_	Yes	Yes	Fujii et al., 2012
Ranitidine	H_2_	No	Unusual	Mauran et al., 2016


The second generation CNS-sparing H_1_ antihistamine, betahistine is not normally known to induce delirium, but an investigation in a side-effects databases did reveal several cases in which delirium may have been present, even though the term, delirium, was not actually used. In this case, delirium was potentially due to the combination of an elevated betahistine plasma level and, significantly, a damaged blood-brain barrier due to cerebral infarctions, confirmed both by computed tomography (CT) and Magnetic resonance imaging (MRI) scans (Hoenders and Wilterdink, 2004). It is noted that caution is often required when prescribing antihistamine H_1_ antagonists for people at risk of delirium and considered individual patient assessment is recommended. In contrast, a small, recent study showed cyproheptadine, a first generation anti-H_1_ antihistamine, with its range of diverse effects was proposed to be a potential option for prevention of postoperative delirium. In this pilot study, cyproheptadine significantly decreased the incidence, but not severity of postoperative delirium (this may relate to its central-permeability). In contrast, the main negative feature of promethazine (another first generation H_1_ antihistamine) is delirium, the probability of which can be predicted from the dose ingested by the individual (Page et al., 2009).

Studies on the association between CNS negative symptoms (psychosis, agitation, hallucinations, mental status changes, disorientation, confusion, irritability, a greatly reduced level of consciousness or hostility all underpinning delirium) and H_2_ blockers have been explored previously. These reactions generally occur during the first 2 weeks of therapy and resolve within 3 days of drug withdrawal, although long term use in critical care or the community may prove problematical. The estimated incidence of CNS negative symptoms is 0.2% or less in outpatients, but significantly higher, 1.6 to 80% in long-term hospitalized patients.

CNS side effects such as mental confusion (major facet of delirium) develop in elderly patients and in patients with severe renal or hepatic impairment. Cimetidine is CNS-permeable in the elderly and critically ill patients (with compromised blood-brain barrier). Cimetidine is frequently associated with these “delirium” reactions; however, no clear evidence exists that one H_2_ blocker is more likely than another to cause such a reaction (Cantú and Korek, 1991). It has been noted that for people at risk of delirium, certain drug combinations are to be prescribed with care. Caution is also required when prescribing antihistamine H_2_ antagonists for people at risk of delirium and a considered individual patient assessment is advocated. Many case studies have been reported to support this policy. One example was a “serious” case of severe mania leading to hospitalization in a 42-year-old alcohol-dependent 4 days after ranitidine introduction (Mauran et al., 2016). Histamine H_2_ receptor antagonists may also cause acute or chronic cognitive impairment. These effects are often associated with some of the H_2_-histamine receptor antagonists, eg., cimetidine (Tagamet) again, but are unusual with ranitidine (Zantac), potentially again due to their respective CNS permeability (Mauran et al., 2016). An old study showed that that a large dose of H_2_-receptor antagonists (50–259 micrograms ivt) decreased hypothalamic NA content (Nowak, 1980). A comparison was made between two groups of patients in a small study who were treated with H_2_ antagonists or proton pump inhibitors (PPI group) for anastomotic ulcer prevention following surgical treatment of esophageal cancer. It was noted that the incidence of delirium was significantly lower in the PPI group than in the H_2_ group. Significantly, in the 11 patients from the H_2_ group who developed delirium, discontinuation of H_2_ antagonists resulted in a significant reduction in the delirium rate score. This study indicated that switch from H_2_ blockers to PPIs reduced delirium and, thus, providing an appropriate strategy to combat drug-induced delirium using antiulcer drugs (Fujii et al., 2012). The ventrolateral preoptic nucleus is a sleep-promoting nucleus located in the basal forebrain. A commonly used intravenous anesthetic, propofol, had been reported to induce sleep and augment the firing rate of neurons in ventrolateral GABAergic preoptic nucleus, but the underlining mechanism is yet to be clearly determined. Interestingly, the propofol-induced inhibition of inhibitory postsynaptic currents on noradrenalin-inhibited neurons have been shown to be mediated by histaminergic H_1_ and H_2_ receptors (Liu et al., 2017).

### Opioids and Histamine Interactions

An interaction between histaminergic and opioidergic systems within the CNS was proposed three decades ago, suggesting that analgesia produced by opioids may be associated with release of histamine and the stimulation of histamine receptors at the supraspinal (central) level (Nishibori et al., 1985). Many more recent studies have shown that histamine receptor antagonists can modulate the analgesic action of opioids, however, the site and mode of action of this interaction differs between the spinal or supraspinal level, and depends on the subtype of histamine receptor (Mobarakeh et al., 2002, 2006, 2009; Stein et al., 2016). A series of studies have also shown than in H_1_R and H_2_R KO mice, morphine-induced antinociception was significantly augmented when compared to the wild-type controls in models of acute pain. Therefore, anti-histamines should be prescribed with caution in people at risk of delirium, but this should be tempered by the observation that untreated severe pain can itself trigger delirium.

### Rationale for Use of H_3_ Antagonists for Future Development?

Cortical activation (EEG desynchronization) is one of the salient signs of wakefulness, attention and enhanced cognitive function, and requires high histaminergic, and cholinergic, noradrenergic, and serotonergic tones, controlled by H_3_ auto- and heteroreceptor action, respectively. Arousal induced by the histamine system through the H_3_ histamine heteroreceptor blockade is believed to largely involve excitation of noradrenergic neurons in the LC. As discussed above, the Histamine H_3_ receptor is expressed on and controls a population of the TM histamine/GABA-ergic neurons which are co-active in parallel and their ascending histamine/GABA fibers release histamine and GABA onto the PFC. Glutamatergic pyramidal neurons in the PFC send excitatory projections back to the histamine neurons in the TMN, reinforcing wakefulness, attention and consciousness. Selective blockade of the H_3_-autoreceptor with an H_3_ receptor antagonist would be predictive to drive this positive reinforcement.

Histamine promotes wakefulness by tonic control over sleep-generating mechanisms in the preoptic/anterior hypothalamus, and cholinergic neurons seem to be implicated. The role of histamine indicates that the histaminergic system also influences attention and learning and memory performance by modulating the release of ACh, although some cognitive effects of histamine and histaminergic agents occur independent of ACh. H_3_R antagonists are well known to enhance cognition and rescue cognitive deficits in preclinical models and modulate neurotransmission (Chazot, 2010), through, in particular, acetylcholine (ACh) release in the cortex and hippocampus, two key brain areas involved in memory processing. It has been recently shown that histamine H_3_ receptor antagonist/inverse agonists require the integrity of brain histamine system to successfully elicit physiological and procognitive effects in the mouse (Provensi et al., 2016). Perfusion of the TMN with the H_3_ inverse agonist/antagonist (ABT-239) differentially increased histamine release from the TMN, NBM, and PFC, but not from the STR or NAcc. When administered locally, ABT-239 (H_3_ receptor antagonist) again increased histamine release from the NBM, but not from the NAcc. As defined by their sensitivity to ABT-239, histaminergic neurons establish distinct pathways according to their terminal projections, and can differentially modulate neurotransmitter release in a brain region-specific manner (Munari et al., 2013; Provensi et al., 2016). This implies independent functions of subsets of histamine neurons according to their terminal projections, with relevant consequences for the development of specific compounds that affect only subsets of histamine neurones, thus increasing target specificity. The selective mode of action is currently believed to be due to the respective levels of presynaptic H_3_Rs expressed on the TMN and cholinergic neurons (Giannoni et al., 2009). This requires formal confirmation. Overall, we provide an anatomical, pharmacological and physiological rationale for developing a CNS-permeable H_3_ histamine receptor antagonist/inverse agonist as a strategy for combatting the range of components of delirium.

## Author Contributions

All authors listed have made a substantial, direct and intellectual contribution to the work, and approved it for publication.

## Conflict of Interest Statement

The authors declare that the research was conducted in the absence of any commercial or financial relationships that could be construed as a potential conflict of interest.
